# Supplementary figures and images for: CXCR4-CXCL12-CXCR7 and PD-1/PD-L1 in Pancreatic Cancer: CXCL12 Predicts Survival of Radically Resected Patients
Source: Cells. 2022 Oct 22;11(21):3340. doi: 10.3390/cells11213340 (PMC9655815; doi:10.3390/cells11213340)

**Supplementary Figure S1.**

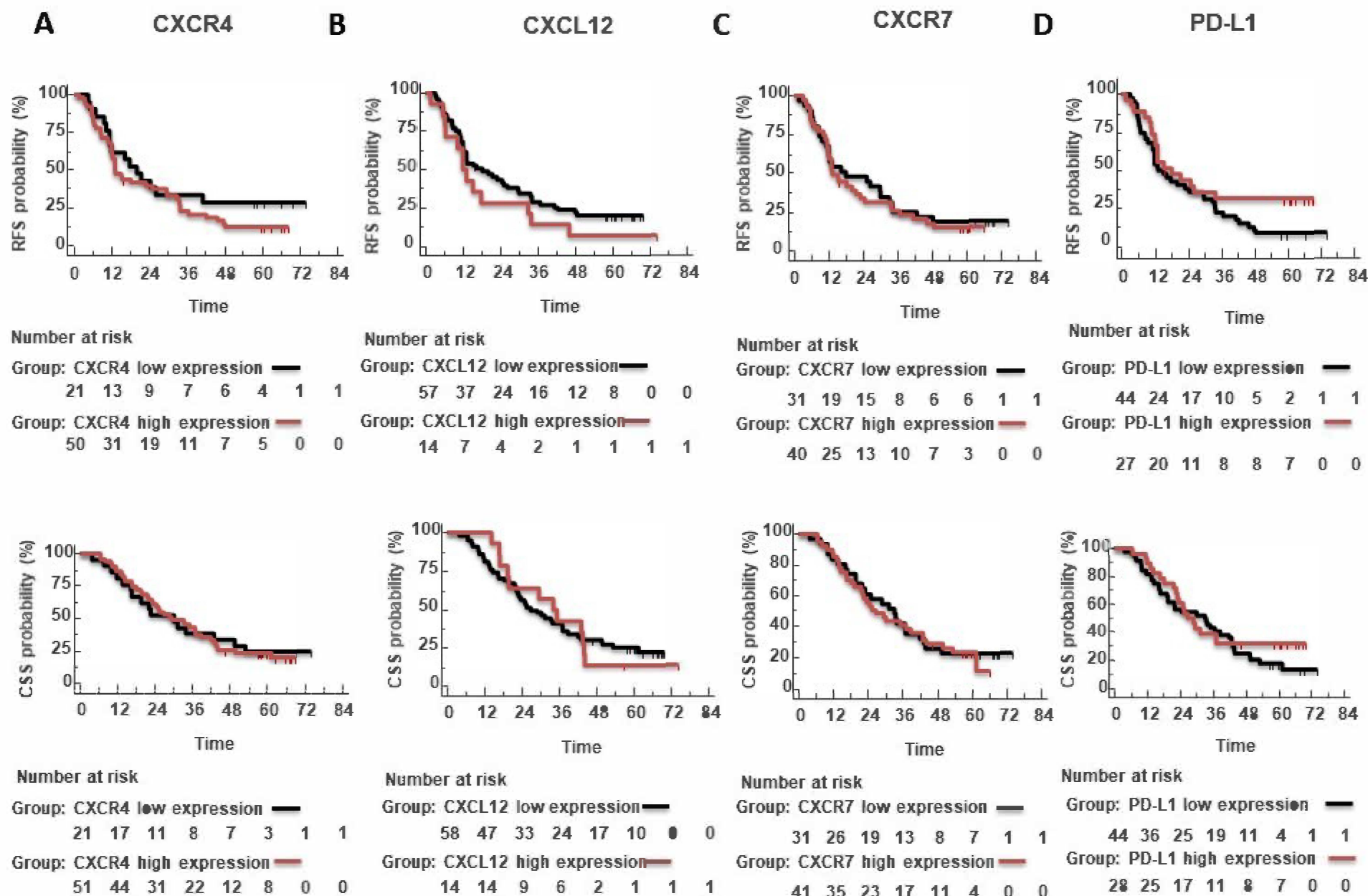

Supplement: Supplementary file 1 [file cells-11-03340-s001.zip › cells-1843323-supplementary/cells-1843323-supplementary.pdf]
